# Structure of Slitrk2–PTPδ complex reveals mechanisms for splicing-dependent *trans*-synaptic adhesion

**DOI:** 10.1038/srep09686

**Published:** 2015-05-19

**Authors:** Atsushi Yamagata, Yusuke Sato, Sakurako Goto-Ito, Takeshi Uemura, Asami Maeda, Tomoko Shiroshima, Tomoyuki Yoshida, Shuya Fukai

**Affiliations:** 1 Structural Biology Laboratory, Life Science Division, Synchrotron Radiation Research Organization and Institute of Molecular and Cellular Biosciences, The University of Tokyo, Tokyo 113-0032, Japan; 2 Department of Medical Genome Sciences, Graduate School of Frontier Sciences, The University of Tokyo, Chiba 277-8501, Japan; 3 CREST, JST, Saitama 332-0012, Japan; 4 Department of Molecular and Cellular Physiology, Shinshu University School of Medicine, Nagano 390-8621, Japan; 5 Institute for Biomedical Sciences, Interdisciplinary Cluster for Cutting Edge Research, Shinshu University, Nagano 390-8621, Japan; 6 Department of Molecular Neuroscience, Graduate School of Medicine and Pharmaceutical Sciences, University of Toyama, Toyama 930-0194, Japan; 7 PRESTO, JST, Saitama 332-0012, Japan

## Abstract

Selective binding between pre- and postsynaptic adhesion molecules can induce synaptic differentiation. Here we report the crystal structure of a synaptogenic *trans-*synaptic adhesion complex between Slit and Trk-like family member 2 (Slitrk2) and receptor protein tyrosine phosphatase (RPTP) δ. The structure and site-directed mutational analysis revealed the structural basis of splicing-dependent adhesion between Slitrks and type IIa RPTPs for inducing synaptic differentiation.

Synapse formation is initiated at contact sites between axon terminals and dendrites, where pre- and postsynaptic adhesion molecules form *trans*-synaptic complexes to induce synaptic differentiation. Presynaptic type IIa receptor protein tyrosine phosphatases (RPTPs) (PTPδ, PTPσ and LAR in mammals) can induce synaptic differentiation by binding to various postsynaptic adhesion molecules such as IL-1 receptor accessory protein-like 1 (IL1RAPL1), IL-1 receptor accessory protein (IL-1RAcP), netrin-G ligand-3 (NGL-3), neurotrophin receptor tyrosine kinases C (TrkC) and Slit- and Trk-like (Slitrk) proteins^1^,^2^,^3^,^4^,^5^,^6^,^7^,^8^. In mammals, the Slitrk family consists of at least six members (Slitrk1-6)^9^,^10^. Mutations in *SLITRK* genes have been reported to be associated with neuropsychiatric disorders^11^,^12^,^13^,^14^. All Slitrk members except Slitrk3 can induce both excitatory and inhibitory synapses^7^,^8^, whereas Slitrk3 selectively induces inhibitory synapses through binding to PTPδ^7^. Therefore, it has been proposed that the selective binding between Slitrk proteins and PTPδ plays a role in balancing excitatory and inhibitory synapses^7^,^8^.

Selective binding between the type IIa RPTPs and their cognate postsynaptic partners is regulated in part by alternative splicing of the type IIa RPTPs^3^,^4^,^6^. PTPδ expressed in the developing brain consists of three immunoglobulin-like (Ig) and four fibronectin type III (Fn) domains in the extracellular region, where two splice sites exist within Ig2 and between Ig2 and Ig3 (Fig. 1a). Peptide insertions (termed mini-exon peptides) at these two splice sites (referred hereafter to as meA and meB) generate splice variants of PTPδ. In this study, we determined the crystal structure between the first leucine-rich repeat (LRR1) of mouse Slitrk2 and the Ig1–Fn1 domains of PTPδ containing both meA and meB. The structure showed that Slitrk2 LRR1 directly recognizes meB but not meA. Further structure-based mutational analyses using surface-plasmon resonance (SPR) spectroscopy and synaptogenic co-culture assay demonstrated that binding of Slitrk2 to PTPδ depends on meB.

## Results and Discussion

### Overall structure

**Figure 1:**
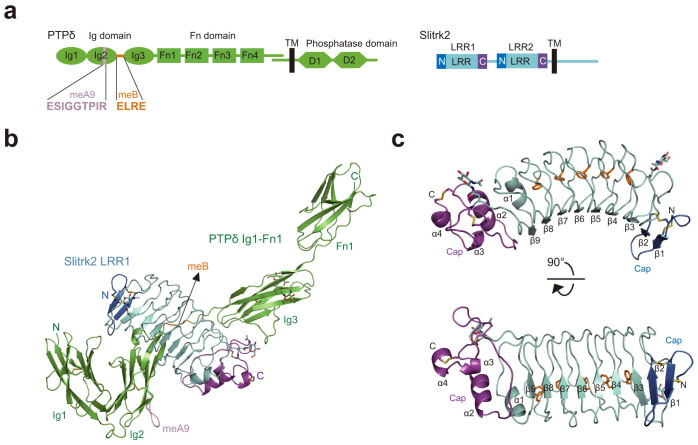
Overall structure of the complex between Slitrk2 LRR1 and PTPδ Ig1-Fn1. (a) Domain architecture of PTPδ and Slitrk2. (b) Overall structure of the complex between Slitrk2 LRR1 and PTPδ Ig1-Fn1. Disulfide bonds and N-linked glycans are shown as sticks. PTPδ Ig1-Fn1 is colored in green, except that the meA and meB insertions are colored in pink and orange, respectively. The N-terminal cap, central LRR and C-terminal cap of Slitrk2 LRR1 are colored in blue, cyan and magenta, respectively. (c) Structure of Slitrk2 LRR1. Disulfide bonds and N-linked glycans are shown as sticks. Phenylalanine residues in the Phe spine structure are shown as orange sticks. The coloring scheme is the same as that in (b).

For structural studies, we initially examined the PTPδ-binding region of Slitrk4 and found that mouse Slitrk4 LRR1 is sufficient for binding to the extracellular domain of PTPδ (PTPδ-ECD) (Supplementary Figure 1a). However, our co-crystallization trials using Slitrk4 LRR1 and the full-length or truncated PTPδ-ECD failed. We then screened other Slitrk members in terms of their expression level in FreeStyle293F cells and their binding activities to PTPδ-ECD and selected mouse Slitrk2 LRR1 as the next candidate for crystallization. After optimization of the length of PTPδ-ECD (Supplementary Figure 1b), we finally determined the crystal structure of a complex between Slitrk2 LRR1 and PTPδ Ig1-Fn1 at 3.35 Å resolution (Fig. 1a,b, Supplementary Figure 2a and Table 1). The asymmetric unit contains one complex, where Slitrk2 LRR1 binds to PTPδ Ig1-Fn1 at a ratio of 1:1 (Fig. 1b, Supplementary Figure 2b). Slitrk2 LRR1 interacts with PTPδ Ig2-3. The overall structure of PTPδ Ig1-Fn1 exhibits an elongated shape. PTPδ Ig1-2 forms a compact V-shaped unit, similarly to LAR or PTPσ Ig1-2^15^. Ig3 is spatially separated from Ig1-2 by meB. The following Fn1 is linearly aligned to Ig3. Slitrk2 LRR1 comprises a central LRR with seven parallel β-strands flanked by N- and C-terminal caps, which are stabilized by disulfide bonds (Fig. 1c). The total nine β-strands in the N-terminal cap and LRR form a concave surface of Slitrk2. The interior of the convex side is rich in completely conserved phenylalanine residues, which form a “Phe spine” structure as observed in the Nogo receptor^16^ (Fig. 1c and Supplementary Figure 3).

### Binding interface

**Figure 2:**
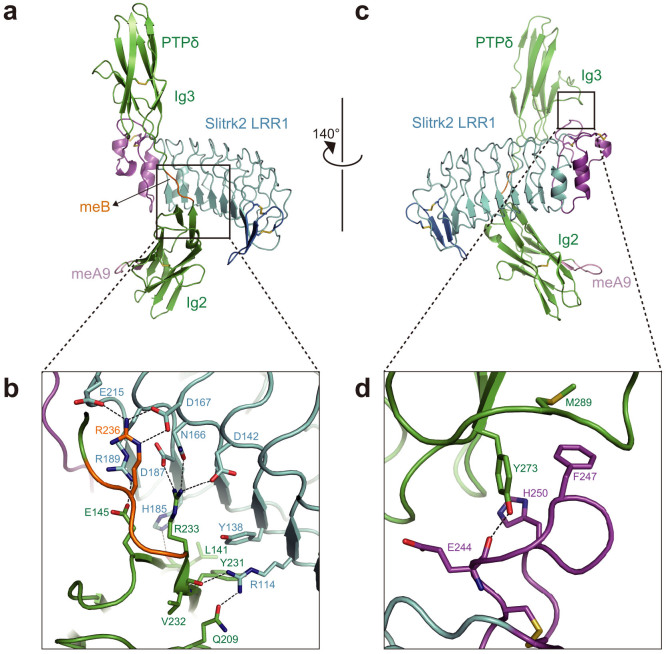
Binding interface between Slitrk2 and PTPδ. (a) Interface between Slitrk2 LRR1 and PTPδ Ig2-meB. The coloring scheme is the same as that in Figure 1. (b) Close-up view of (a). (c) Interface between Slitrk2 LRR1 and PTPδ Ig3. The coloring scheme is the same as that in Figure 1. (d) Close-up view of (d).

The concave surface of Slitrk2 LRR1 surrounds two strands in PTPδ Ig2 and meB (^234^ELRE^237^) with a buried surface area of 644 Å^2^ (Fig. 2a). Asp167 and Glu215 of Slitrk2 hydrogen bond with Arg236 of PTPδ (the third residue of meB) (Fig. 2b). In addition, Asp142, Asn166 and Asp187 of Slitrk2 hydrogen bond with Arg233 of PTPδ (one-residue upstream of meB). Surrounding these interactions, Arg114 and Arg189 of Slitrk2 hydrogen bond with Gln209 and Glu145 of PTPδ, respectively, whereas Arg114 and His185 of Slitrk2 hydrogen bond with the main-chain O atoms of PTPδ Val232 and Leu141, respectively. Furthermore, Tyr138 of Slitrk2 is stacked with Tyr231 of PTPδ. These Slitrk2 residues involved in the interaction with PTPδ Ig2-meB are mostly conserved in all mouse Slitrk members (Supplementary Figure 3), suggesting that they can potentially recognize PTPδ Ig2-meB in the same manner as Slitrk2.

Slitrk2 LRR1 also interacts with PTPδ Ig3 with a buried surface area of 450 Å^2^ (Fig. 2c). Phe247 and His250 of Slitrk2 hydrophobically interact with Tyr273 of PTPδ, which hydrogen bonds with the main-chain O atom of Slitrk2 Glu244 (Fig. 2d). Phe247 of Slitrk2 also hydrophobically interacts with Met289 of PTPδ and appears to play a central role in the Slitrk2 LRR1–PTPδ Ig3 interface. However, this phenylalanine residue is replaced by Thr, Ser or Pro in Slitrk1, 4 or 6, respectively (Supplementary Figure 3), which are inadequate to form hydrophobic interactions with Tyr273 and Met289 of PTPδ. In addition, His250 of Slitrk2 is not conserved in Slitrk1, 4 or 6 (Supplementary Figure 3). Therefore, the observed Slitrk2–PTPδ Ig3 interactions may be specific to Slitrk2, 3 and 5.

### Splicing-dependent interactions and synaptogenic activity

**Figure 3:**
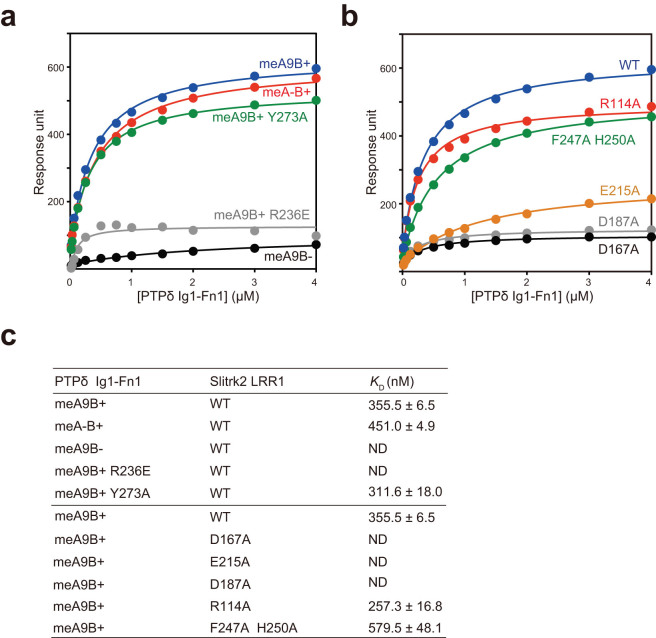
Recognition of PTPδ meB by Slitrk2 LRR1. (a) SPR equilibrium analysis of the interaction between Slitrk2 LRR1 and the splicing variants or mutants of PTPδ Ig1-Fn1. Slitrk2 LRR1 was immobilized on a CM5 sensor tip and the indicative PTPδ Ig1-Fn1 proteins were injected. Response units were plotted against concentrations of the indicative PTPδ Ig1-Fn1 proteins. (b) SPR equilibrium analysis of the interaction between Slitrk2 LRR1 mutants and PTPδ Ig1-Fn1 (meA9/meB+). (c)Dissociation constants calculated from (a) and (b).

**Figure 4:**
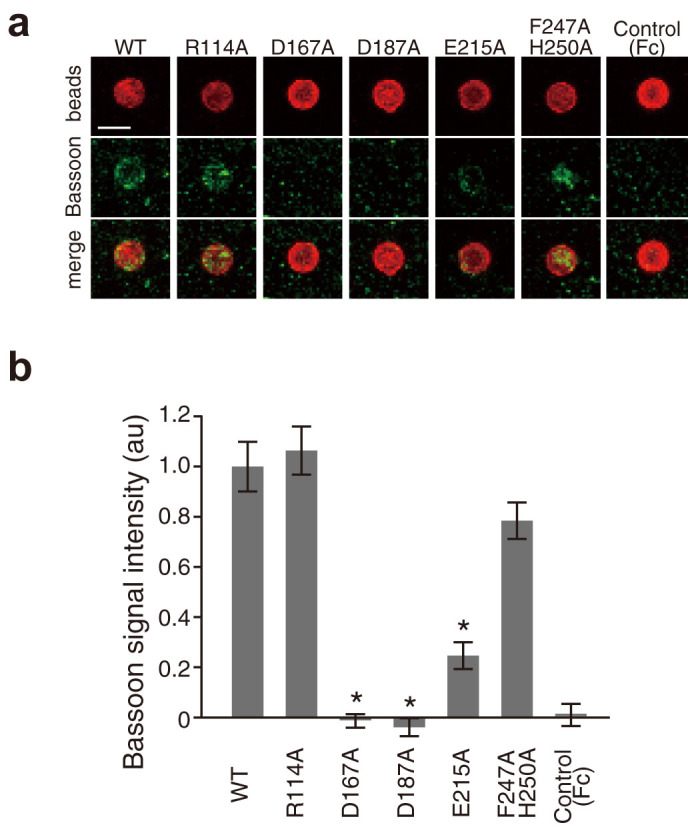
Synaptogenic activities of Slitrk2 LRR1 mutants. (a) Co-cultures of cortical neurons and beads conjugated with Fc or Slitrk2 LRR1 mutants fused to Fc. Slitrk2-induced presynaptic differentiation was monitored by immunostaining of the co-cultures with antibodies against Fc (red) and presynaptic active zone protein Bassoon (green). Scale bar, 5 μm. (b) Staining signal intensities for Bassoon on beads conjugated with Fc or Slitrk2 LRR1 mutants fused to Fc. All values represent as mean ± SEM. * p < 0.0001, compared to wild-type Slitrk2-LRR1-Fc. n = 24–29 beads.

In the crystal structure of the Slitrk2–PTPδ complex, Slitrk2 LRR1 directly recognizes the meB insertion of PTPδ, whereas the meA insertion is distant from Slitrk2 LRR1 (Fig. 2a). Therefore, the present structure clearly indicates that binding of PTPδ to Slitrk2 depends on meB but not on meA. To ensure this finding, we examined the binding of Slitrk2 LRR1 to the PTPδ variants containing either or both meA and meB (meA9B-, meA-B+ and meA9B+) by surface-plasmon resonance (SPR) spectroscopy (Fig. 3a). As expected, the meA9B+ and meA-B+ variants bound to Slitrk2 LRR1 with similar affinities, whereas the meA9B- variant hardly bound to Slitrk2 LRR1 (Fig. 3a, c and Supplementary Figure 4). We next assessed the observed Slitrk2–PTPδ interactions by site-directed mutagenesis (Fig. 3 and Supplementary Figure 4). The R236E mutation of PTPδ and the D167A and E215A mutations of Slitrk2, which disrupt the meB-specific interactions, abolished the binding between Slitrk2 and PTPδ. The D187A mutation of Slitrk2, which disrupts the hydrogen bond with Arg233 of PTPδ (one-residue upstream of meB), also abolished the binding. Accordingly, the D167A, D187A or E215A mutant of Slitrk2 abolished or significantly reduced their synaptogenic activities in our co-culture assay (Fig. 4). On the other hand, the R114A mutation of Slitrk2 (PTPδ Ig2-mediated interface), the Y273A mutation of PTPδ or the F247A H250A double mutation of Slitrk2 (PTPδ Ig3-mediated interface) hardly affected the binding. Therefore, the R114A or F247A H250A mutant of Slitrk2 exhibited the synaptogenic activity comparable to wild-type Slitrk2 (Fig. 4). These results perfectly support the finding that binding of PTPδ to Slitrk2 depends on meB (Fig. 3 and Supplementary Figure 4).

### Structural comparison with the Slitrk1 LRR1–PTPδ complex

Recently, the crystal structure of the complex between Slitrk1 LRR1 and PTPδ Ig1-3 has been reported^17^. The main-chain structure of Slitrk1 LRR1 is almost identical to that of Slitrk2 LRR1 with an rmsd of 1.3 Å (Supplementary Figure 5a). Slitrk1 LRR1 interacts with PTPδ Ig2-meB in a similar manner to Slitrk2 LRR1 (Supplementary Figure 5b and c). The hydrophilic interactions between PTPδ Ig2-meB and Slitrk LRR1 are essentially the same, consistent with the sequence conservation between Slitrk1 and 2 (Supplementary Figure 3). On the other hand, the relative position and orientation of PTPδ Ig3 to PTPδ Ig2-meB–Slitrk LRR1 are substantially different between the complexes of Slitrk1 and 2 (Supplementary Figure 5d). Concomitantly, the PTPδ Ig3-mediated interfaces are different between the Slitrk1 and 2 complexes (Supplementary Figure 5e). Physiological significance of this difference remains obscure, because the PTPδ Ig3-mediated interface appears unlikely to contribute to the affinity to Slitrk proteins as mentioned above.

*Trans*-synaptic clustering of the Slitrk1–PTPδ complex has been proposed on the basis of the lateral interaction between neighboring complexes in the crystal^17^. This lateral interaction involves Arg72, Phe74 and Arg143 of Slitrk1 and Glu237 and Glu286 of PTPδ. On the other hand, no higher-order clustering was observed in the crystal of the Slitrk2–PTPδ complex (Supplementary Figure 2b). Arg143 of Slitrk1 corresponds to Ser147 of Slitrk2, which is exposed to the solvent (Supplementary Figure 5f). Arg72 and Phe74 of Slitrk1 correspond to Arg76 and Tyr78 of Slitrk2, respectively, which interact with Asp378 of the neighboring PTPδ Fn1 but appear unable to form higher-order clustering (Supplementary Figure 5f).

In conclusion, we revealed that binding of Slitrk2 to PTPδ depends on meB but not on meA, based on the crystal structure and mutational studies at the molecular and cellular levels. Our previous analysis of cDNA from the developing mouse brain showed that PTPσ and LAR also have the splicing variants containing a four-residue insert as meB with the conserved third arginine residue^4^, suggesting that the meB-containing variants of all type IIa RPTPs can potentially bind to all Slitrk proteins. In fact, a recent study showed that the meB-containing variant of LAR can bind to Slitrk1 (ref. 17). Other example of splicing-dependent regulation of *trans*-synaptic adhesion through the type IIa RPTPs is the adhesion between PTPδ and IL1RAPL1 or IL-1RAcP^3^,^4^. Further studies for these complexes, together with the present study, will lead to complete understanding of splicing-dependent regulation of *trans*-synaptic adhesions for synaptic differentiation.

## Methods

### Protein expression and purification

The gene encoding mouse PTPδ Ig1-Fn1 (residues 28–418) was amplified from cDNA (accession No. NM_011211.3) by PCR and cloned into pEBMulti-Neo vector (Wako Pure Chemical Industries) with the N-terminal signal sequence derived from pHLsec vector^18^ and a C-terminal hexahistidine tag. The gene encoding mouse Slitrk2 LRR1 was cloned into pEBMulti-Neo vector with a C-terminal hexahistidine tag. The proteins were transiently expressed in Freestyle293F cells and purified by Ni-affinity chromatography. Slitrk2 LRR1 was further purified by size-exclusion chromatography with Superdex200 increase (GE healthcare).

### Crystallization and structure determination

We tested the interaction between the various truncation mutants of mouse Slitrk proteins and PTPδ by pull down experiments using Fc-fused PTPδ. Consequently, we found that first LRR domain (LRR1) of Slitrk proteins and PTPδ extracellular regions containing Ig1-Fn1 are sufficient for their binding. Slitrk2 LRR1 and PTPδ Ig1-Fn1 were mixed at a molar ratio of 1:1. Prior to the crystallization, the sample was treated with neuraminidase for trimming *N*-linked glycans. Crystallization was carried out with the sitting drop vapor diffusion method at 293 K by mixing the equal volumes (1 μL) of the protein solution and the mother liquor containing 15% PEG4000, 0.1 M sodium acetate, 0.1 M MES (pH 6.0). The crystals were flash-frozen in liquid nitrogen after soaking in the mother liquor containing 25% ethylene glycol for cryoprotection.

Diffraction data were collected at 100 K at BL41XU in SPring-8 and processed with HKL2000^19^. The structure of Slitrk2 LRR1 and PTPδ Ig1-Fn1 was solved by the molecular replacement method using the program MolRep^20^. For PTPδ Ig1-Fn1, Ig1-2 of PTPδ (PDB ID:2YD6), Ig3 of PTPσ (PDB ID:2YD9) and Fn2 of LAR (PDB ID:2DJU) were used as the search model. For Slitrk2 LRR1, the Nogo receptor LRR domain (PDB ID:1OZN) was used as the search model. Model building was carried out using the program Coot^21^. The model was refined using the program Phenix^22^ to the final *R*_work_/*R*_free_ factors of 23.46/28.65%. The stereochemistry of the final model was assessed by Procheck^23^. Data collection and refinement statistics were summarized in Table 1. The buried surface area was calculated using the program PISA^24^. All structural figures are prepared using the program PyMol (Schrödinger, LLC).

### SPR analysis

SPR experiments were carried out by using Biacore T200 (GE healthcare) at 25°C in 10 mM HEPES-Na buffer (pH 7.9) containing 150 mM NaCl and 0.05% Tween-20. The wild-type or mutant Slitrk2 LRR1 (D167A, D187A, E215A, R114A or F247A H250A) was immobilized on a CM5 sensor tip by the amine-coupling method. The splicing variant (meA9B+, meA9B- or meA-B+) or mutant (R236E or Y273A) of PTPδ Ig1-Fn1 was injected at concentrations ranging from 15.625 to 4,000 nM. 10 mM NaOH was used as a regeneration buffer.

### Synaptogenic assay

Primary cortical cultures were prepared from mice at E18 essentially as described previously^4^. Expression vectors for the mutated forms of Slitrk2-LRR1-Fc were generated by PCR-based mutagenesis using pEB6-Slitrk2-LRR1-Fc as a template. Fc and the mutated forms of Slitrk2-LRR1-Fc in FreeStyle293F cell culture medium were bound to Protein A-conjugated magnetic particles (smooth surface, 4.0–4.5 μm diameter; Spherotech). Beads coupled with Fc or the Fc fusion proteins were added to cortical neurons at days in vitro 16. After 24 hours, cultures were fixed for immunostaining with mouse anti-Bassoon antibody (Stressgen, 1:400) and rabbit anti-human Fc (Rockland, 1:1000).

### Image acquisition and quantification

Images of bead-neuron co-cultures were collected from at least two separate experiments. Image acquisition and quantification for the co-culture assays were performed essentially as previously described^4^. Briefly, the intensities of immunostaining signals for Bassoon were measured as the optical mean density within a circle of 7-μm diameter enclosing coated-bead. Statistical significance was evaluated by one-way ANOVA followed by post hoc Tukey’s test.

## Author Contributions

A.Y., A.M., T.S., T.U. and T.Y. performed gene cloning, protein purification and crystallization. A.Y., S.F., Y.S. and S.G.-I. collected the diffraction data sets. A.Y. and S.F. analyzed the collected data and determined the structures. T.S. performed SPR measurement. A.Y. and S.F. wrote the paper with editing by T.U. and T.Y., S.F. designed and supervised the study.

## Additional information

**How to cite this article**: Yamagata, A. *et al.* Structure of Slitrk2–PTPδ complex reveals mechanisms for splicing-dependent *trans*-synaptic adhesion. *Sci. Rep.* 5, 9686; DOI:10.1038/srep09686 (2015).

## Supplementary Material

Supplementary Information Supplementary Figures 1-5

## Figures and Tables

**Table 1:** Data collection and refinement statistics

Slitrk2 LRR1–PTPδ Ig1-Fn1	
**Data collection**	
Wavelength (Å)	1.0000
Resolution (Å)^a^	50.0–3.35 (3.41–3.35)
Space group	*P*2_1_2_1_2_1_
Cell dimensions	
* a*, *b*, *c* (Å)	87.2, 91.3, 123.4
* α*, *β*, *γ* (°)	90, 90, 90
No. of unique reflections	14,509
Completeness (%)^a^	99.2 (97.2)
*R*_sym_ (%)a,b	13.0 (40.7)
*I*/*σΙ*^a^	14.3 (1.9)
Redundancy^a^	9.5 (5.9)
**Refinement**	
No. of atoms	
Protein	4891
NAG	56
*R*_work_/*R*_free_ (%)^c^	23.46/28.65
R.m.s.d.	
Bond lengths (Å)	0.005
Bond angles (°)	1.113
Average B-factor	
Protein	109.6
NAG	111.2
Ramachandran plot	
Most favored (%)	83.6
Disallowed (%)	0.0

^a^Values in parentheses are for the highest-resolution shell.

^b^*R*_sym_ = Σ*_hkl_*Σ*_i_* |*I_i_*(*hkl*) - <*I*(*hkl*)>|/Σ*_hkl_*Σ*_i_* *I_i_*(*hkl*), where *I_i_*(*hkl*) is the *i*-th intensity measurement of reflection *hkl*, including symmetry-related reflections, and <*I*(*hkl*)> is the average.

^c^*R*_work_ = ∑*_hkl_*|*F*_obs_–*F*_calc_|/∑*_hkl_*|*F*_obs_| for work set, and *R*_free_ = ∑*_hkl_*|*F*_obs_–*F*_calc_|/∑*_hkl_*|*F*_obs_| for test set, which comprises randomly-selected 5% of the total reflections.

## Notes

Accession codes: Coordinates and structure factors of the Slitrk2 LRR1–PTPδ Ig1-Fn1 complex have been deposited in the Protein Data Bank under accession code 4Y61.

## References

[b1] Takahashi H. & Craig A. M. Protein tyrosine phosphatases PTPδ, PTPσ, and LAR: presynaptic hubs for synapse organization. Trends Neurosci. 36, 522–534 (2013). 23835198 10.1016/j.tins.2013.06.002 PMC3789601

[b2] Um J. W. & Ko J. LAR-RPTPs: synaptic adhesion molecules that shape synapse development. Trends Cell Biol. 23, 465–475 (2013). 23916315 10.1016/j.tcb.2013.07.004

[b3] Yoshida T. *et al.* Interleukin-1 receptor accessory protein organizes neuronal synaptogenesis as a cell adhesion molecule. J. Neurosci. 32, 2588–2600 (2012). 22357843 10.1523/JNEUROSCI.4637-11.2012 PMC6621890

[b4] Yoshida T. *et al.* IL-1 receptor accessory protein-like 1 associated with mental retardation and autism mediates synapse formation by *trans*-synaptic interaction with protein tyrosine phosphatase δ. J. Neurosci. 31, 13485–13499 (2011). 21940441 10.1523/JNEUROSCI.2136-11.2011 PMC6623287

[b5] Woo J. *et al.* *Trans*-synaptic adhesion between NGL-3 and LAR regulates the formation of excitatory synapses. Nat. Neurosci. 12, 428–437 (2009). 19252495 10.1038/nn.2279

[b6] Takahashi H. *et al.* Postsynaptic TrkC and presynaptic PTPσ function as a bidirectional excitatory synaptic organizing complex. Neuron 69, 287–303 (2011). 21262467 10.1016/j.neuron.2010.12.024 PMC3056349

[b7] Takahashi H. *et al.* Selective control of inhibitory synapse development by Slitrk3-PTPdelta trans-synaptic interaction. Nat. Neurosci. 15, 389–398 (2012). 22286174 10.1038/nn.3040 PMC3288805

[b8] Yim Y. S. *et al.* Slitrks control excitatory and inhibitory synapse formation with LAR receptor protein tyrosine phosphatases. Proc. Natl. Acad. Sci. U. S. A. 110, 4057–4062 (2013). 23345436 10.1073/pnas.1209881110 PMC3593915

[b9] Aruga J. & Mikoshiba K. Identification and characterization of Slitrk, a novel neuronal transmembrane protein family controlling neurite outgrowth. Mol. Cell. Neurosci. 24, 117–129 (2003). 14550773 10.1016/s1044-7431(03)00129-5

[b10] Proenca C. C., Gao K. P., Shmelkov S. V., Rafii S. & Lee F. S. Slitrks as emerging candidate genes involved in neuropsychiatric disorders. Trends Neurosci. 34, 143–153 (2011). 21315458 10.1016/j.tins.2011.01.001 PMC3051006

[b11] Abelson J. F. *et al.* Sequence variants in SLITRK1 are associated with Tourette's syndrome. Science 310, 317–320 (2005). 16224024 10.1126/science.1116502

[b12] Zuchner S. *et al.* SLITRK1 mutations in trichotillomania. Mol. Psychiatry 11, 887–889 (2006). 17003809 10.1038/sj.mp.4001898

[b13] Piton A. *et al.* Systematic resequencing of X-chromosome synaptic genes in autism spectrum disorder and schizophrenia. Mol. Psychiatry 16, 867–880 (2011). 20479760 10.1038/mp.2010.54 PMC3289139

[b14] Smith E. N. *et al.* Genome-wide association of bipolar disorder suggests an enrichment of replicable associations in regions near genes. PLoS Genet. 7, e1002134 (2011). 21738484 10.1371/journal.pgen.1002134 PMC3128104

[b15] Coles C. H. *et al.* Proteoglycan-specific molecular switch for RPTPσ clustering and neuronal extension. Science 332, 484–488 (2011). 21454754 10.1126/science.1200840 PMC3154093

[b16] He X. L. *et al.* Structure of the Nogo receptor ectodomain: a recognition module implicated in myelin inhibition. Neuron 38, 177–185 (2003). 12718853 10.1016/s0896-6273(03)00232-0

[b17] Um J. M. *et al.* Structural basis for LAR-RPTP/Slitrk complex-mediated synaptic adhesion. Nat. Commun. 5, 5423 (2014). 25394468 10.1038/ncomms6423

[b18] Aricescu A.R., Lu W.. & Jones E.Y. A time- and cost-efficient system for high-level protein production in mammalian cells.. Acta. Crystallogr. D 62, 1243–50 (2006). 17001101 10.1107/S0907444906029799

[b19] Otwinowski Z. & Minor W. Processing of X-ray Diffraction Data Collected in Oscillation Mode. Methods Enzymol. 276, 20 (1997). 10.1016/S0076-6879(97)76066-X 27754618

[b20] Vagin A. & Teplyakov A. MOLREP: an automated program for molecular replacement. J. Appl. Crystallogr. 30, 4 (1997).

[b21] Emsley P. & Cowtan K. Coot: model-building tools for molecular graphics. Acta Crystallogr. D Biol. Crystallogr. 60, 2126–2132 (2004). 15572765 10.1107/S0907444904019158

[b22] Adams P. D. *et al.* The Phenix software for automated determination of macromolecular structures. Methods 55, 94–106 (2011). 21821126 10.1016/j.ymeth.2011.07.005 PMC3193589

[b23] Laskowski R. A., Macarthur M. W., Moss D. S. & Thornton J. M. PROCHECK: a program to check the stereochemical quality of protein structures. J. Appl. Crystallog. 26, 9 (1993).

[b24] Krissinel E. & Henrick K. Inference of macromolecular assemblies from crystalline state. J. Mol. Biol. 372, 774–797 (2007). 17681537 10.1016/j.jmb.2007.05.022

