# Supplementary material for: Structure of Slitrk2–PTPδ complex reveals mechanisms for splicing-dependent trans-synaptic adhesion
Source: Sci Rep. 2015 May 19;5:9686. doi: 10.1038/srep09686 (PMC4437028; doi:10.1038/srep09686)
Supplement: Supplementary Information — Supplementary Figures 1-5 [file srep09686-s1.pdf]

Structure of Slitrk2–PTPδ complex reveals mechanisms for splicing-dependent *trans*-synaptic adhesion

Atsushi Yamagata[1,2,3], Yusuke Sato[1,2,3], Sakurako Goto-Ito[1,3], Takeshi Uemura[3,4,5], Asami Maeda[1,3], Tomoko Shiroshima[1,3], Tomoyuki Yoshida[6,7]* & Shuya Fukai[1,2,3]*

[1]Structural Biology Laboratory, Life Science Division, Synchrotron Radiation Research Organization and Institute of Molecular and Cellular Biosciences, The University of Tokyo, Tokyo 113-0032, Japan

[2]Department of Medical Genome Sciences, Graduate School of Frontier Sciences, The University of Tokyo, Chiba 277-8501, Japan

[3]CREST, JST, Saitama 332-0012, Japan

[4]Department of Molecular and Cellular Physiology, Shinshu University School of Medicine, Nagano 390-8621, Japan

[5]Institute for Biomedical Sciences, Interdisciplinary Cluster for Cutting Edge Research, Shinshu University, Nagano 390-8621, Japan

[6]Department of Molecular Neuroscience, Graduate School of Medicine and Pharmaceutical Sciences, University of Toyama, Toyama 930-0194, Japan

[7]PRESTO, JST, Saitama 332-0012, Japan

*Correspondence should be addressed to e-mail: fukai@iam.u-tokyo.ac.jp or toyoshid@med.u-toyama.ac.jp

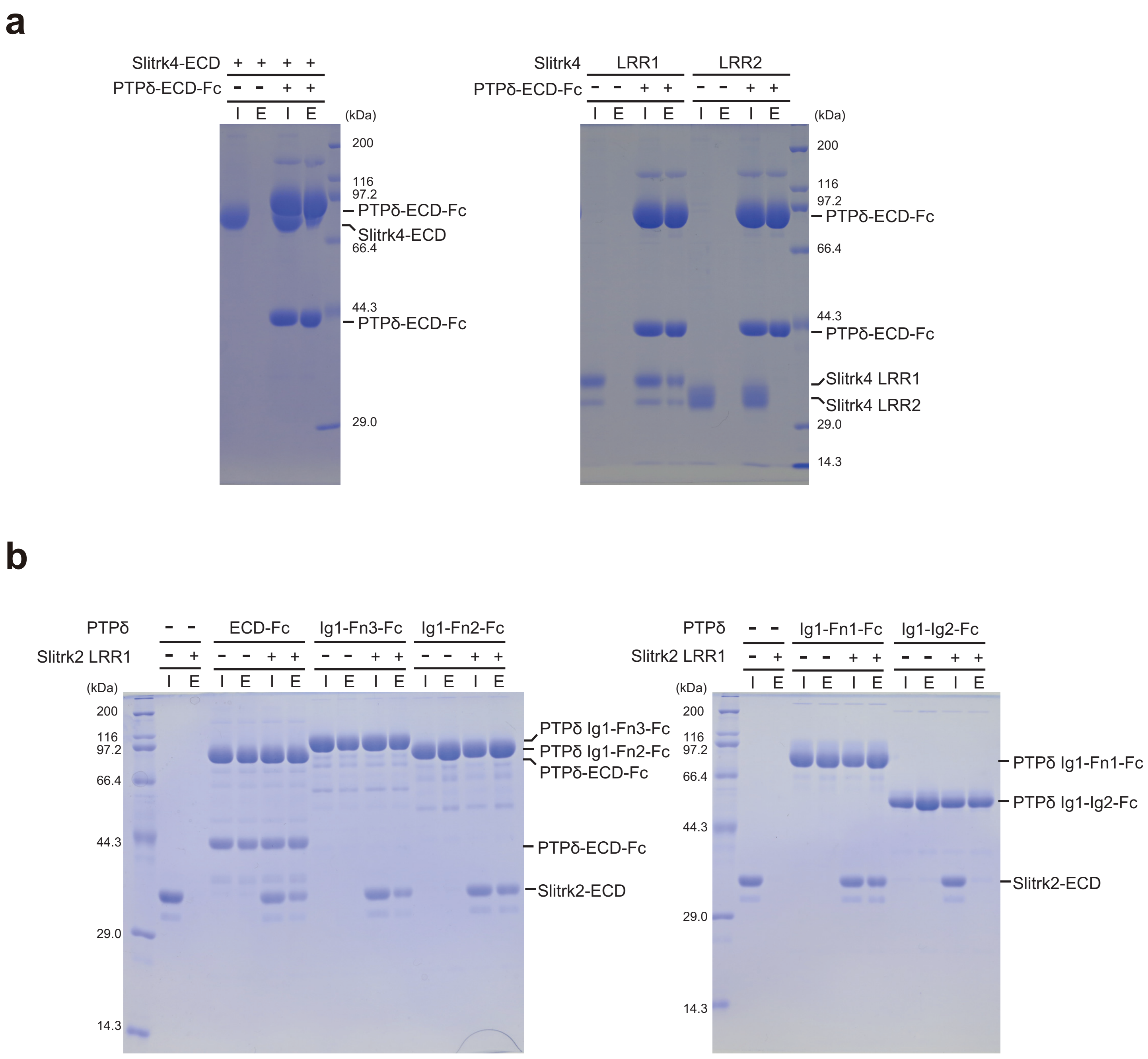

Supplementary Figure 1. Characterization of binding between PTPδ and Slitrk4 or 2

(a) Deletion analysis of Slitrk4-ECD for binding to PTPδ-ECD. PTPδ-bound Slitrk4 proteins were resolved by SDS-PAGE without boiling and stained by Coomassie Brilliant Blue.

(b) Deletion analysis of PTPδ-ECD for binding to Slitrk2 LRR1. Slitrk2 LRR1-bound PTPδ proteins were resolved by SDS-PAGE without boiling and stained by Coomassie Brilliant Blue. Lanes I and E indicate the input and the elution, respectively. Note that the C-terminal region of PTPδ-ECD (corresponding to the lower-molecular-weight band) is non-covalently attached to the rest of PTPδ-ECD (corresponding to the higher-molecular-weight band).

a

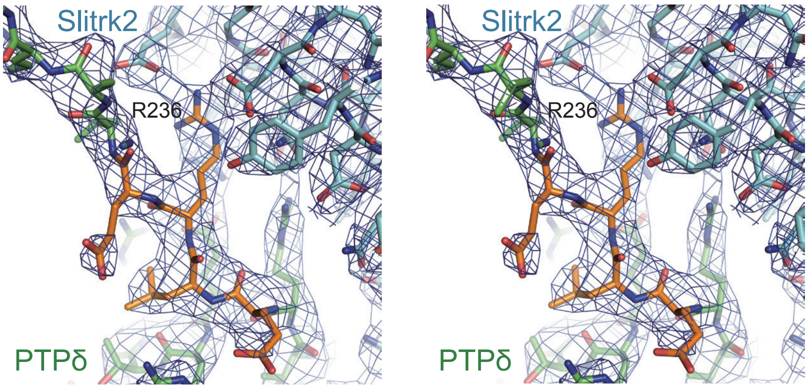

b

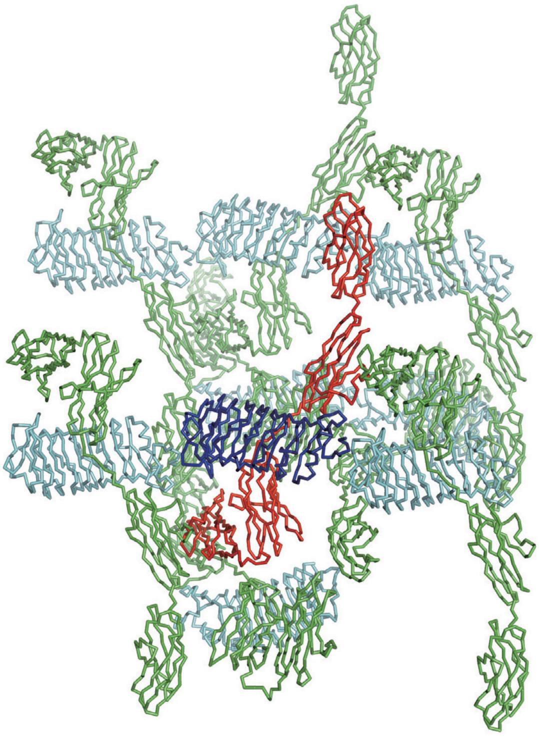

Supplementary Figure 2. Electron density map and crystal packing

(a) $2F_o$-F_c electron density map around meB, contoured at 1.2 σ level (stereo view). PTPδ is colored in green, except that meB is colored in orange. Slitrk2 is colored in cyan.

(b) Crystal packing. Slitrk2 LRR1 and PTPδ Ig1-Fn1 in one complex are colored in blue and red, respectively, while those in their crystallographic symmetry-related complexes are colored in cyan and green, respectively.

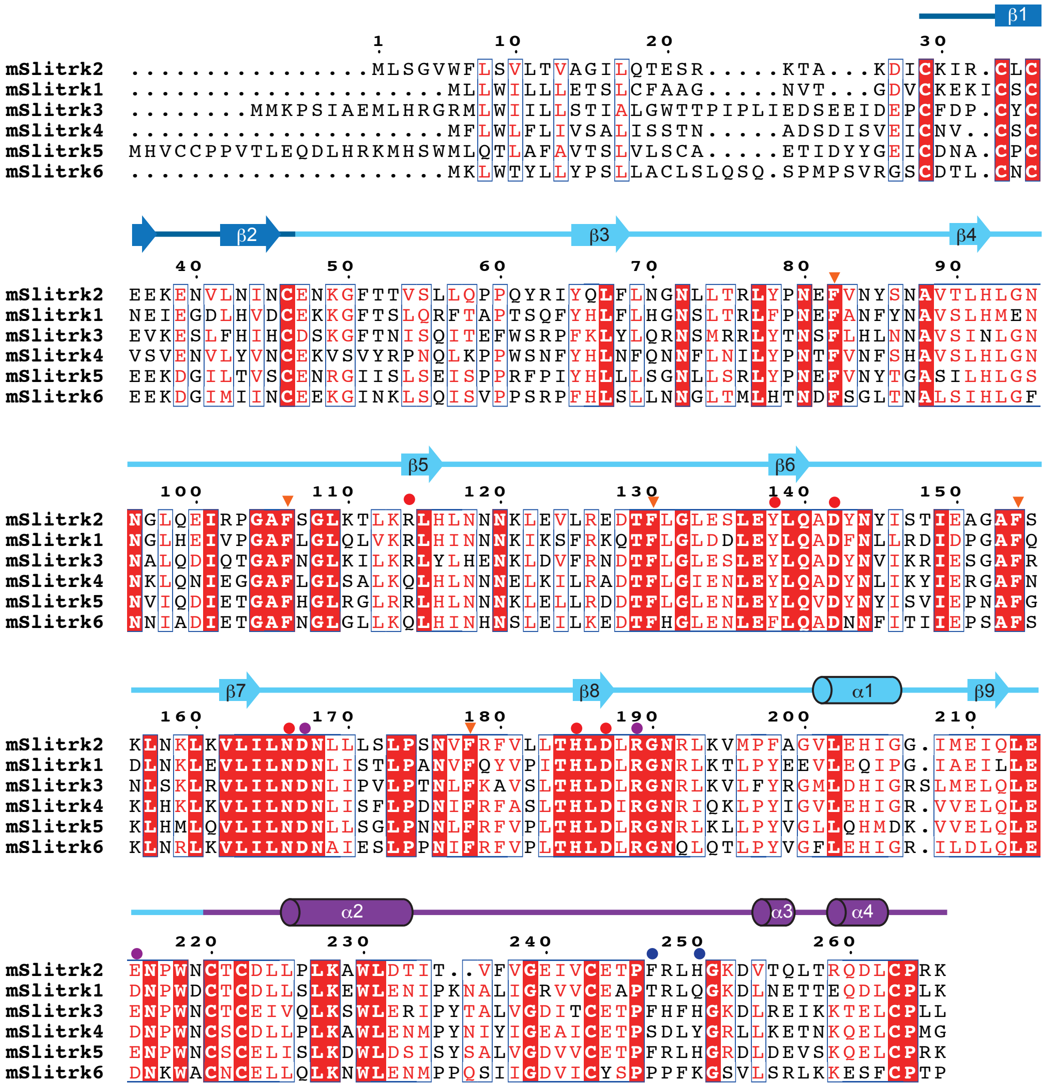

Supplementary Figure 3. Sequence alignment of Slitrk LRR1

The amino acid sequences of LRR1 of Slitrk1-6 from *Mus musculus* are aligned. The secondary structure of Slitrk2 LRR1 is shown above the sequence. The coloring scheme is the same as that in Fig. 1c. The Slitrk2 residues that interact with PTPδ meB, Ig2 or Ig3 are indicated by magenta, red or blue circles, respectively. Phenylalanine residues in the Phe spine are indicated by orange reverse triangles.

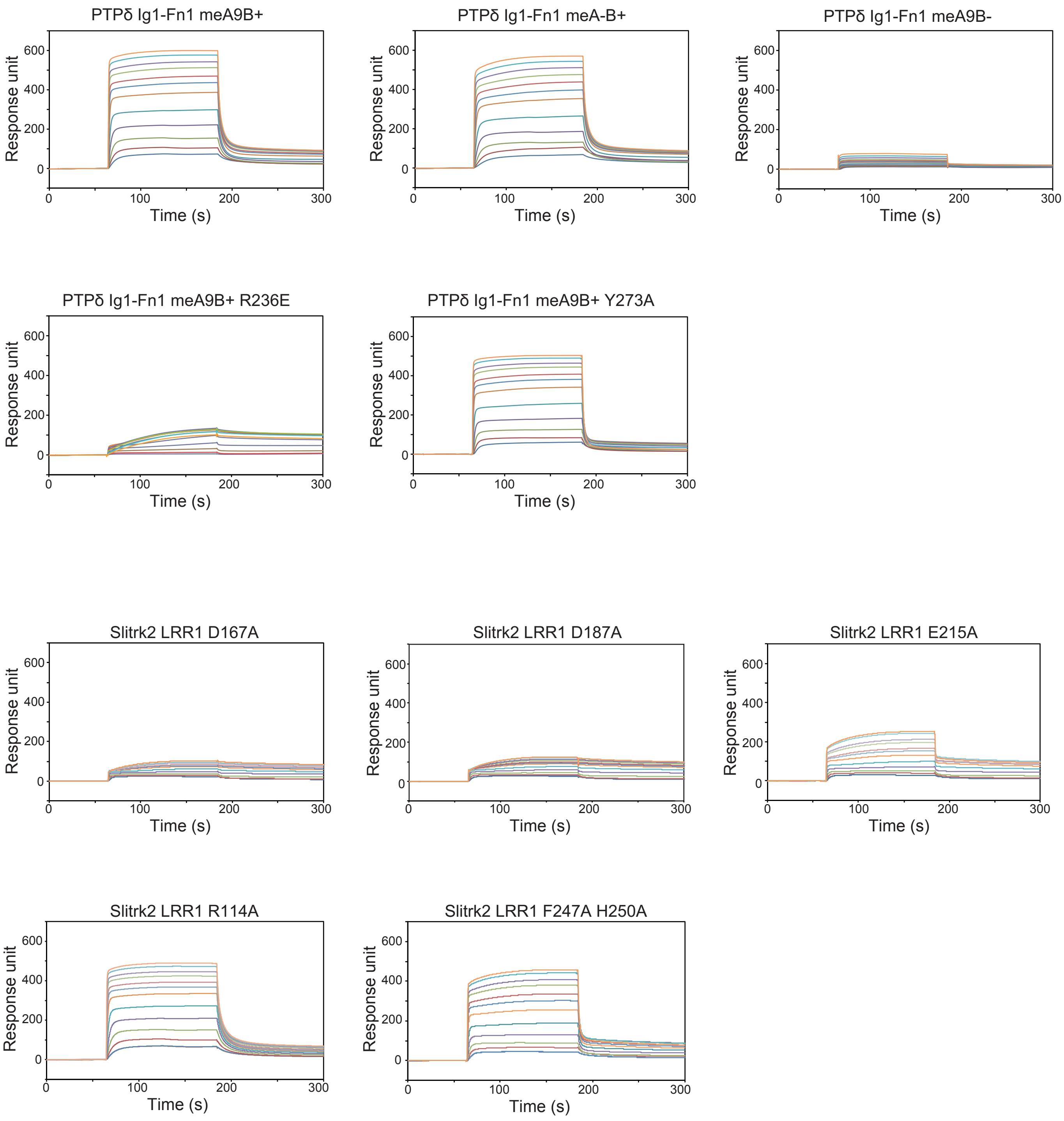

Supplementary Figure 4. SPR sensorgrams for analyzing the interaction between Slitrk2 LRR1 and PTPδ Ig1-Fn1

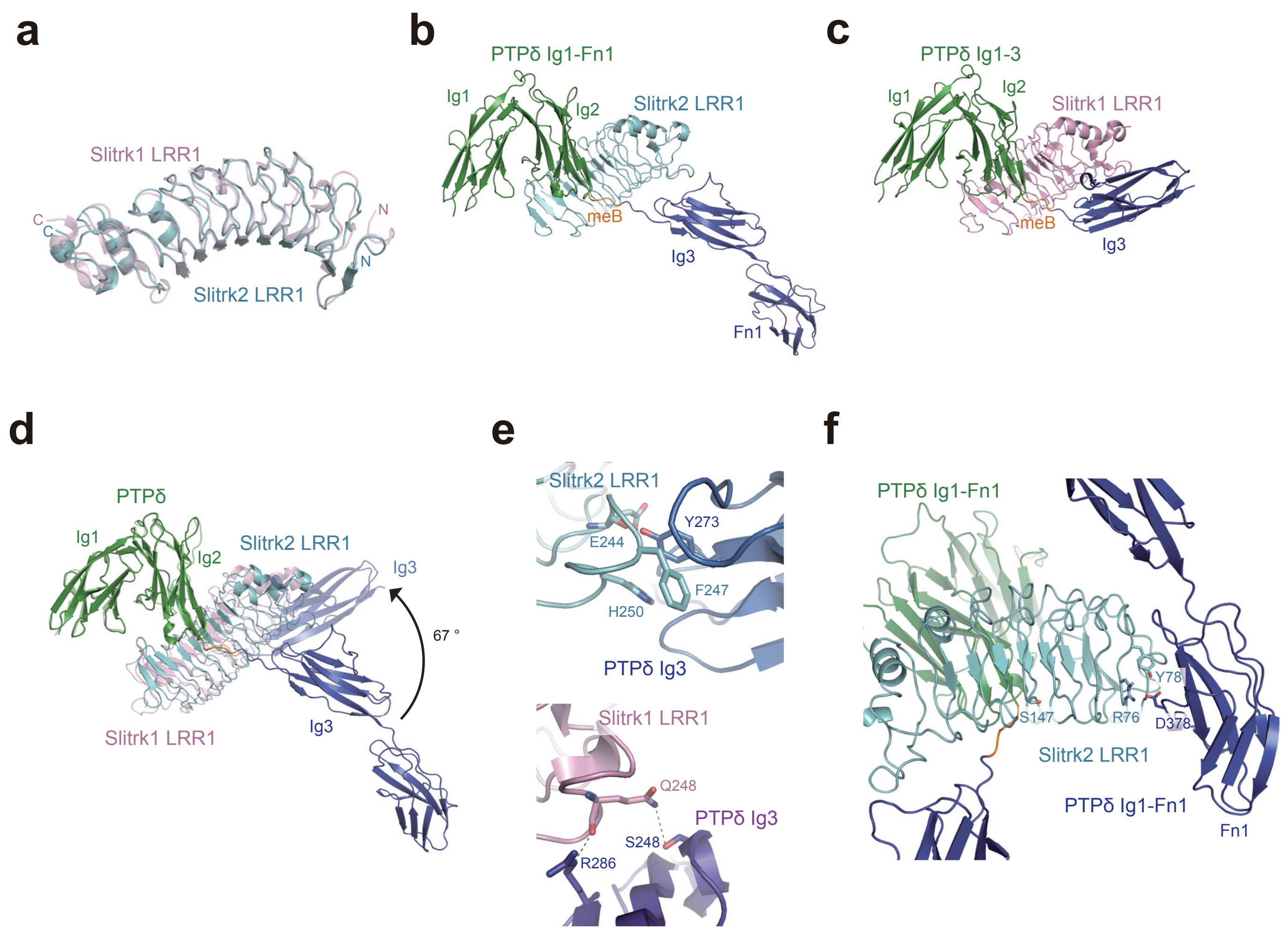

Supplementary Figure 5. Structural comparison with the Slitrk1–PTPδ complex

(a) Superposition of Slitrk1 LRR1 on Slitrk2 LRR1. Slitrk1 LRR1 and Slitrk2 LRR1 are colored in pink and cyan, respectively.

(b) Structure of PTPδ Ig1-Fn1 in complex with Slitrk2 LRR1. Ig1-2 and Ig3-Fn1 of PTPδ are colored in green and blue, respectively. Slitrk2 LRR1 is colored in cyan.

(c) Structure of PTPδ Ig1-3 in complex with Slitrk1 LRR1 (PDB ID:4RCA). The coloring scheme is the same as that in (b), except that Slitrk1 LRR1 is colored in pink.

(d) Superposition of the Slitrk1-bound PTPδ on the Slitrk2-bound PTPδ, using PTPδ Ig1-2 (green) as the reference. The Ig3 domain in the Slitrk1-bound PTPδ (pale blue) and that in the Slitrk2-bound PTPδ (deep blue) can be related to each other by 67˚ rotation.

(e) Difference in the Ig3-mediated interface between the Slitrk2–PTPδ complex (upper panel) and the Slitrk1–PTPδ complex (lower panel). The interacting residues are shown as sticks. The coloring scheme is the same as that in (c).

(f) Crystal contacts between neiboring Slitrk2–PTPδ complexes. The coloring scheme is the same as that in (b).
